# Factors affecting utilization of health facilities for labour and childbirth: a case study from rural Uganda

**DOI:** 10.1186/s12884-019-2674-z

**Published:** 2020-01-16

**Authors:** Jaya A. R. Dantas, Debra Singh, May Lample

**Affiliations:** 1 International Health Programme, School of Public Health, Curtin University, Kent Street, Bentley, Perth, WA 6102 Australia; 2 Kimanya-Ngeyo Foundation for Science and Education, Plot no. 51, Main Street, Apartment 1, 1600 Jinja, Uganda; 3 The Office of Public Affairs of the Baha’is of the United States, 1320 19th Street, NW, Washington, DC 20036 USA

**Keywords:** Antenatal care, Male involvement, Maternal health outcomes, Uganda

## Abstract

**Background:**

Since 2000 considerable attention has been placed on maternal health outcomes as the 5th Millennium Goal. In Uganda, only 65% of births are delivered by a skilled birth attendant, contributing to the 435 women that die in every 100,000 births from unattended complications. Factors that impact a women’s decision on where to deliver include cost and household barriers, poor health services and lack of education.

**Methods:**

Insight into factors impacting maternal health decision-making in two villages in South Eastern Uganda, were explored through a cross-sectional study using focus group discussions (FDGs) with men and women and administering a simple questionnaire.

**Results:**

For men and women in the villages, cultural and community patterns of behavior have the strongest impact on delivery options. While women with no complications could often find options to deliver safely, lack of emergency obstetric care remains a strong factor in maternal deaths.

**Conclusions:**

This article proposes that communities be engaged in identifying and leveraging their strengths to find solutions for challenges facing women in achieving safe deliveries.

## Introduction

Approximately 75% of births worldwide occur outside of a health facility. In these cases the presence of skilled birth attendants is uncommon, with most deliveries attended by a relative or a Traditional Birth Attendant (TBA) and in some cases, no one at all [1]. This can contribute to complications and maternal death. Globally, three quarters of maternal deaths are caused by postpartum bleeding, sepsis, eclampsia, obstructed labour and complications of unsafe abortions. The vast majority of obstetric complications are in developing countries [1], mostly during time of delivery and cannot necessarily be predicted during antenatal care. Therefore it is important that women deliver with a skilled birth attendant who can refer their patient to a hospital in the event of an emergency [2].

The situation in Uganda is similar to that of many resource poor developing countries. Over the last decade, maternal mortality in Uganda has decreased from 500/100,000 to 360/100, 000 in 2013 [3]. Trained health workers attended 65% of births, but 35% are attended by relatives or TBAs [4, 5]. According to a 2012 report from the WHO, 81% of pregnant women had at least one antenatal visit but only 61% had the four recommended by the WHO [5]. In addition to poorly trained and inadequate numbers of health workers, most health facilities in Uganda, at all levels, lack basic equipment and infrastructure, running water, electricity and operating theatres and are not equipped to offer emergency care [6].

In 1996, the Ministry of Health, along with the United Nations Population Fund (UNFPA) and Uganda Population Secretariat launched a project that focused on a means of referral during emergency maternal health cases and concentrated mainly on communication, transport and quality of health services delivery [4]. Despite reproductive health interventions such as equipping ambulances with VHF radio capability to allow communication with the TBAs, only 11.7% of women deliver in a fully functional emergency obstetric care facility; despite the fact that 15% of all pregnancies develop complications that require emergency care [7].

Approximately 53% of women deliver with trained health professionals in facilities that do not have access to blood, oxygen or perform emergency caesarean sections, and the remainder birth at home [8]. These statistics, coupled with the high rate of fertility in Uganda, with an average of seven children per woman, increases the chances of morbidity and mortality [9]. In Uganda, as in most developing countries, the main causes of maternal mortality can be attributed to the three delays: the delay to seek care, the delay to reach facilities and institutional delay to provide care [10]. The factors that contribute to these delays are discussed below, structured under the headings of Access, Availability and Acceptability.

### Access

#### Distance and cost barriers

Distance to the health centre has proven to be a major deterrent in women seeking both antenatal care as well as delivery with a skilled birth attendant [11, 12]. Families and women who cannot afford to pay for transportation to a health facility, are unable to access this care if the facility is not within walking distance. There is also the issue of having to provide consumable such as soap and blankets at the clinic [13], even in free government facilities in Uganda. Availability of funds, or lack thereof, can often determine whether women attend antenatal care or deliver in a health centre, with women from higher income families more likely to utilize health facilities for birth [14]. While the husband, and sometimes, independently, the wife, will attempt to put money aside in advance, other emergencies or just basic needs often use up the savings.

#### Status of women

The status of women in Uganda is such that the husband is usually the primary decision-maker. Men decide issues such as the timing of sexual relations, family size and whether the wife will use health services [11, 15, 16]. Men also control the household finances and in cases where they are absent for any period it may either cause considerable delay or prevent the timely seeking of health care [16]. Delays in seeking care also occur when women wait until late in the birth process to seek out a skilled birth attendant. Many women report they birthed at home due to a ‘quick labour’, where the women believe the stages of delivery progressed too quickly to reach a health centre [17].

#### Educational factors

Women who have higher education levels are more likely to seek antenatal care and deliver at the clinic rather than with a TBA [14]. Furthermore, educated women have the opportunity to work and have a higher income, which in turn impacts their nutritional status and potentially reduces complications. Educated women are also more likely to limit the number of children thereby reducing their chance of life threatening maternal health issues [18]. Young, unmarried girls, who hide their pregnancy and may not have partner support, have difficulty negotiating safe deliveries [14].

### Availability

#### Health professionals

One of the greatest obstacles to maternal health is the limited availability of skilled maternal health professionals [11, 16]. Absenteeism of health professionals is common, and may be attributed to their heavy workloads, low-resource settings and low remuneration which force them to undertake income-generating activities outside health work [16]. Furthermore, maternal health care is impeded by health professionals whose knowledge and skills are inadequate. This results in inappropriate management of complications and untimely care in clinics and hospitals. Women have at times given birth in hospitals alone because of negligence [12].

Vogel et al. (2016) through the application of mixed methodologies investigated the barriers and facilitators to implementation of World Health Organization (WHO) maternal health recommendations in Uganda, and other lower-income countries [19]. Shortages in the health workforce was identified as one of the key barriers that limited provision and delivery of high-quality care, in Uganda specifically, poor recruitment and retention rates especially in rural areas. Furthermore, quotas on recruitment of health care workers at various facilities was identified as a barrier [19].

#### Health infrastructure

The 20 year civil war, the brutal regimes of Idi Amin and Milton Obote II, resulted in damage to all infra-structure and a lack of development in most of Uganda with significant impacts in Northern Uganda for nearly two decades [11]. Even where basic infrastructure has been rebuilt the health care workers face issues of poor flow of equipment and medications, delays in salaries, issues of housing and schooling for their children resulting in high absentee rates [20]. They are frequently overwhelmed by the numbers of patients and lack the tools to adequately diagnose and treat the multiple and complex cases that present to them This study was conducted in two villages in Eastern Uganda which also has limited facilities beyond the main town of Jinja.

### Acceptability

Provider attitudes are also a major hindrance to utilization of health facilities [12]. Many rural women who deliver in hospitals face ridicule because of poverty, sub-standard clothing, poor personal hygiene, and cries of pain. At times procedures are undertaken without consulting the mother who is left feeling confused and disrespected [21]. These issues often deter women from seeking care at clinics [12]. One 2013 study [16] found that mothers had concerns about their treatment at health clinics. Women feared being mistreated by midwives and birth attendants, and had experienced slapping, pinches and derogatory remarks. Women unable to provide their own soap, blankets and baby clothes due to poverty felt humiliated [12]. Questions were raised regarding cultural sensitivities as women were treated with lack of dignity [16, 22]. Women complained about feeling rushed by midwives and doctors to deliver within a certain time frame and often having their own cultural wishes denied, such as upright birthing positions and having a trusted family member present [16].

One study found that when birthing in health centres, women felt they had to suppress expressions of pain which traditionally the women considered to be part of delivery [23]. However, other researchers dispute this cultural belief and report a cultural practice of not vocalising during labour for fear of harming the baby, or demonstrating strength [11, 16]. Complaints about government health facilities also included staff giving inaccurate information, expectations of bribes and abandonment during labour [12]. These negative feelings towards health facilities mean that women largely only turn to these services in cases of emergency [16, 23].

The sense of mistrust is present among healthcare workers as well, who felt that women’s ignorance and refusal to use healthcare facilities during labour contributed to high levels of maternal mortality. While acknowledging that these health facilities are not always fully equipped to contend with the needs of women, they criticized women for not understanding why they needed to be referred to hospitals [16]. In contrast, women, particularly in rural areas, have a particular trust in TBAs, who have often played an important role in local childbirth for many years and are considered an integral part of motherhood. Strong cultural preferences for birthing at home where valued cultural practices can be followed are also prevalent, and it is difficult for women to disregard that history and place their trust in skilled birth professionals in health centres [11, 12, 16].

### Poor infrastructure

Women were also concerned about birthing conditions, including hygiene, water supplies and privacy [22]. In a study that looked at the birthing environments at the home and facilities found that only a third of women experienced child delivery in Uganda where the environment had ‘improved water’ (as defined by incidences constructed by study investigators) [23, 24]. Moreover, across Uganda and Eastern Africa, less than 50% of birthing facilities had improved water and sanitation [24]. A number of key documents have highlighted the need to increase resources in global maternity care, including the improved training of health professionals, the provision of suitable housing for those professionals and the building of adequate facilities to enable high quality care to be provided [25, 26].

With this background, factors that impact a women’s decision on where to deliver include cost and household barriers, poor health services and lack of education. This qualitative case study explored the factors impacting maternal health decision-making in two villages in South Eastern Uganda, study using focus group discussions (FDGs) and a simple questionnaire.

## Methods

### Research design

This cross-sectional exploratory case study using was undertaken in two villages in the southeastern region of the country, Maligita and Kibibi. Both villages are about 30 km outside of Jinja (on opposite sides of the Nile River and both approximately 6 km from the nearest Health Centre IV). Maligita has the presence of a trained community midwife, as well as the support of a faith-based organization that assists with antenatal care and immunizations. Kibibi which has no private clinic, instead relies on the presence of the Health Centre II and IV (See Table 1)**.** Since specific data about obstetric outcomes is not available, the villages were chosen because they would both face similar challenges in terms of distance from emergency care during labour and the second author lived and worked as a medical doctor for a decade near the villages.

**Table 1:** Health Centre categories in Uganda

Infrastructure Level	Administrative Level	Target Population	Services Provided
Health Centre I	Village	1000	Community based health care prevention, VHTs^a^
Health Centre II	Parish	5000	Preventative, curative care, outreach
Health Centre III	Sub-county	20,000	Preventative, curative, outreach, inpatient, maternity, labouratory services
Health Centre IV	County	100,000	Preventative, curative, outreach, inpatient, blood transfusion and surgical care

^a^ VHTs are made up of volunteer community health workers that provide preventative health education to community members

This study used a simple demographic questionnaire with some open-ended questions, in Luganda (one of the local languages spoken in the area), FGDs and community key informant interviews and field work combined with observations were undertaken. Luganda is the predominant language spoken in Maligita, while both Luganda and Lusoga are spoken in Kibibi. Lusoga is less of a written language than Luganda. Prior to our FGDs, we used key informants to pilot the questions. The key informants were the community midwife and a Community Health Worker (CHWs). CHWs in Uganda are generally lay individuals who have received a few days of training. They work with one to nine others in their village to make up the Village Health Team (VHT). While they are trained in a number of areas, they mostly operate through campaigns that respond to the perceived needs by the government. Through their feedback, questions were modified in order to fit the cultural context.

### Participant recruitment

Participants were recruited through our key informants using purposive and snowball sampling. In Maligita, the community midwife and a local VHT member helped bring participants for the focus group, served as translators and assisted participants with filling in the questionnaire. In Kibibi, the CHW verbally informed village residents about the study prior to its commencement by making visits to leaders, home visits within the village and announcements at antenatal clinics. As such, a large number of people who met the inclusion criteria were present when the research team arrived. The decision was made not to turn people away. In Kibibi, a local VHT member as well as a local resident helped organize FGDs and assisted with the translation. The inclusion criteria for both villages required individuals to be pregnant or have had children in the past 1–3 years. In appreciation for their participation, people were given a drink and a small snack. All participants were over 18 years.

### Focus group discussion

In Maligita there were three FGDs: the first with women that had given birth recently or who had small children, the second with women who were currently pregnant and finally with men whose wives were pregnant or had given birth in the past year. There were five or six participants in each focus group. In Kibibi, there were two FGDs: women and men who had young children. There were 10–12 people in each focus group in Kibibi.

The FGDs in both villages were moderated by the second author with the assistance of a key informant, while recording and transcription were undertaken by the first author. The questionnaires were either self-administered where the participant was sufficiently literate or with the assistance of a research assistant if needed. The research assistant was fluent in both Luganda and Lusoga, and therefore able to explain the questions when needed. Since he was involved in translating the questions from English to Luganda and in field testing the questionnaires he was thoroughly acquainted with the questions prior to the research.

A total of 35 participants across the two villages completed questionnaires immediately prior to the FGDs. In Maligita, questionnaires were administered to: 1. Pregnant women as they visited the antenatal clinic run by the community based, certified midwife (CM); 2. Women who had delivered within the past three years; and 3. Men whose wife were pregnant or had delivered within the past year. In Kibibi, it was only women who had delivered within the past three years; and men whose wife were pregnant or had delivered within the past year as there was no equivalent CM in Kibibi to run antenatal clinics.

### Ethical considerations

The Human Research Ethics Committee at Curtin University in Western Australia approved our study. Each of the participant was given a participant information sheet in Luganda and provided informed consented via a signature or thumb print. The participant information sheet was read to the participant if they were sub-literate and translated to Lusoga where needed. Participants were informed that they could withdraw from the study at any time if required. Participants were also assured of anonymity and were assigned a pseudonym.

### Analysis

The data from interviews and FGDs was recorded and in addition was transcribed and translated with pseudonyms attached to each respondent. A multidisciplinary team (a medical doctor, maternal and child health graduate student and a health sociologist) undertook thematic analysis looking for emerging patterns related to the research questions in the data. A six stage process was used: we familiarised ourselves with the data, generated initial codes, searched for themes among codes, reviewed the themes, defined and named themes, and wrote the report [27]. Themes from the FGD analysis were categorized under “antenatal care”, “preparation for birth”, “male involvement” to reflect the interviewees’ statements. We then discussed emerging categories and drew themes and interpretations on the basis of group consensus. Standard descriptive statistics (frequencies and percentages) were used to summarize the responses to the demographic data and close-ended questionnaire questions.

The following snapshots of the two villages provide a glimpse of the village community and an overview of the context in which the study was undertaken:

### Unique features of Kibibi village

Kibibi is located in the sub-county of Budondo. Budondo has unusually high rates of delivery in health facilities compared to the rest of the country. The Local Chairperson at the sub-county level recently completed a Master’s degree in Safe-Motherhood and has a high level of commitment to improving outcomes in this area in the sub-county. His Master’s research found that there were 88% of women delivering in facilities (personal communications and sighting of the Master’s thesis) compared to a national average 57% [8]. The women in Kibibi gave high priority to delivering either at the Health Centre IV or the main hospital in the district. There was also an obvious sense of community support, whereby neighbors helped one another to find funds at the time of delivery if shortage of funds was an issue. Women felt that it was important to be safe during childbirth. (Information was provided by the CHWs in the village). An environmental scan revealed that participants in Kibibi appear to have more financial means. Women from Kibibi, during the focus groups also appeared less dependent on their husbands and had access to their own financial means through small village businesses.

### Unique features of Maligita village

The community midwife, CM has lived in Maligita since 2000 after coming to Jinja and seeing a woman on the street, in labour, who needed help. She stayed with the woman, helped her give birth and then realized there was a great need for skilled birth attendants. Through the help of midwives from overseas she was able to build a health centre in the village and began to teach pregnant women and administer antenatal care in hers and neighbouring villages.

In the event of an emergency, the CM refers women to the hospital and, if possible, accompanies them to ensure quality of care. She advocates for the women and ensures that care is provided in a timely fashion, wherever possible. In information shared by the CM, she stated that although she does not receive payment from the women, the women give her some token of appreciation, as cash if they can, in kind or produce. She is greatly valued in the surrounding community. She has developed a trusting and caring relationship in the community, is available 24 h and will go beyond the call of duty to ensure that a woman whose baby she is delivering has a good outcome. The village is along a main road, but the village appeared to have less means than Kibibi and the women had fewer possibilities of having access to independent finances.

### Common antenatal practices

In both Kibibi and Maligita antenatal care includes providing women with a “passport” in which is marked their immunization history, whether they have received a mosquito net, their weight, prenatal vitamins and position of the fetus. A physical examination is also done, including taking blood pressure, measuring the women’s fundal height and listening to the fetal heartbeat. There is also postnatal care in which babies are weighed and given immunizations. During antenatal care women are counselled on family planning and preparing for labour. They are encouraged to save funds for transportation in case of emergencies as well as the acquisition of a mother kit.

## Results

Tables 2 and 3 below provide an overview of participant characteristics in each village of the study:

**Table 2:** Summary of participant characteristics – Maligita village

	Male		Female		Total	
	N	%	N	%	N	%
Religion						
Christian	4	67%	7	54%	11	58%
Muslim	2	33%	6	46%	8	42%
Marital Status						
Single	0	0%	2	15%	2	11%
Married	6	100%	10	76%	16	84%
Divorced	0	0%	1	8%	1	5%
Widowed	0	0%	0	0%	0	0%
Age						
18–28	2	33%	8	62%	10	53%
29–39	1	17%	5	38%	6	32%
40–50	2	33%	0	0%	2	10%
Over 50	1	17%	0	0%	1	5%
Number of Children						
1–3	2	33%	8	62%	10	53%
4–7	2	33%	4	30%	6	32%
8–11	2	33%	1	8%	3	16%
N/A	0	0%	0	0%	0	0%
Education						
Primary	2	33%	6	46%	8	42%
Secondary	4	67%	4	30%	8	26%
Post-secondary	0	0%	1	8%	1	5%
None	0	0%	2	15%	2	11%
Education status						
Studying	0	0%	0	0%	0	0%
Left school	6	100%	11	85%	17	88%
Never went	0	0%	2	15%	2	11%

**Table 3:** Summary of participant characteristics – Kibibi village

	Male		Female		Total	
	N	%	N	%	N	%
Religion						
Christian	0	0%	4	40%	4	25%
Muslim	6	100%	6	60%	12	75%
Marital Status						
Single	0	0%	1	10%	1	6%
Married	6	100%	9	90%	15	94%
Divorced	0	0%	0	0%	0	0%
Widowed	0	0%	0	0%	0	0%
Age						
18–28	0	0%	8	80%	8	50%
29–39	3	50%	2	20%	5	31%
40–50	3	50%	0	0%	3	19%
Over 50	0	0%	0	0%	0	0%
Number of Children						
1–3	0	0%	4	40%	4	25%
4–7	4	67%	6	60%	10	63%
8–11	1	16%	0	0%	1	6%
N/A	1	16%	0	0%	1	6%
Education						
Primary	1	16%	6	60%	7	44%
Secondary	5	83%	4	30%	8	50%
Post-secondary	0	0%	0	0%	0	0%
None	0	0%	0	0%	0	0%
Education status						
Studying	0	0%	0	0%	0	0%
Left school	6	100%	10	100%	16	100%

Of the 12 women interviewed from Kibibi, all had visited a health centre or hospital during the pregnancy and delivered there with the exception of one who delivered at home. Almost all of the women in Maligita, except for one who was referred to a hospital by CM, delivered with the midwife at the clinic. The focus groups and questionnaires revealed the following as influencing their choices:

### Access

The women in Kibibi did not mention money as a hindrance to reaching the Health Centre IV, although the men touched on this. With the distance to the health centre being 6 km and the hospital being 30 km away, it is not possible to walk, especially if the women are in labour. However, the women explained that if they lack funds, they are able to approach their neighbours who would offer as much money as they could. Only one of the women mentioned the husband as being the factor in deciding where to give birth.

The women from Maligita did, however, mention that a lack of transport and funds made it difficult for them to reach the nearest hospital. The hospital expects them to have a mother kit containing gloves, a razor and covering for the bed, while this is not always possible due to lack of funds. Their transport to the hospital is a motorcycle taxi (boda boda), assuming they have the money to be able to do so, which many do not. With CM, women can give as much as they can afford and at times provide alternative to money such as food. For most, CM’s clinic is within walking distance in the village.

### Availability

In Kibibi, women had access to both private and public facilities. There was, however no registered midwife, nor private facilities similar to those of Maligita. However, CM’s clinic in Maligita, through faith-based donations, had access to water, electricity and a private space for women to deliver. This provided the women with a viable alternative to government clinics.

### Acceptability

This issue of unprofessional health-workers, or “bad character” of health professionals was a thread that was repeated many times, by all participants, in both the questionnaires and FGDs.

“At health centre IV, they are there but they are very harsh and rude.”

(Kibibi women’s focus group)

“When you go with money, they are quick... Because you don’t pay them they go slowly slowly.” (Maligita pregnant women’s focus group).

Several mothers in Kibibi mentioned specifically that they went to the hospital because that is where the other mothers around them went and because they knew they would have good quality of care. They had access to HC II and III which could be reached by foot, but they mostly bypassed these facilities, because they were perceived as inadequate. Many women said, stated that:

“The health workers there were harsh, proud and at times slow to care for them. They knew it was their best option in case of emergency and that seemed to override their problems with health staff.” (Kibibi women’s focus group).

Several women explained that once CM moved into their neighborhood, they no longer felt any need to go to the Health Centre IV and contend with the problems they often encountered there. They outlined proximity and very good quality of care as being the two reasons they preferred to visit the private clinic:

“Once CM (midwife) came, no more government hospital.” (Maligita women post labour focus group).

“Clinic is easier, it is near and they are serious, better than government, the government clinic is rude, they are proud.” (Maligita pregnant women’s focus group).

For the women who participated in this study, the delays that impacted their seeking healthcare stemmed more from difficulties at the institutional level than from their own initiative. The majority of the women actively sought care and had the opportunity to do so. Furthermore, they knew the potential dangers of not seeking care.

All of the women except five knew someone who had died during labour. One woman remarked, “Many mothers die in labour, we are getting used to it.” They identified poor quality of care, multiple deliveries, lack of blood, giving birth without a skilled attendant, distance, ignorance, position of baby, husband’s neglect and lack of funds and transport as being the primary causes for maternal mortality.

Of the 11 men interviewed for this study, 10 of their wives delivered with a nurse/midwife, and one with a TBA. The men identified distance, good service, healthcare personnel and confidence in care as being the key determinants in where a woman decides to give birth. Additionally, they identified lack of antenatal care, poor healthcare workers, distance to health centre as being with primary causes of maternal mortality, with one man identifying youth as a factor and another a big baby. Overall, in Kibibi, it was considered normal to deliver in a Health Centre IV, therefore, women preferred this as their first option. Maligita benefits from a trained midwife, available 24 h, have an established relationship with her and feel comfortable to turn to her for delivery.

## Discussion

This study explored the factors affecting utilization of health facilities for antenatal care and childbirth in two villages in Eastern Uganda. The findings around transport and cost issues reflect that of previous studies, however, participants shared new information about women’s education and birth choices. The literature on reproductive health indicates that women who are educated are more likely to deliver with a skilled birth attendant [14]. However, in our study despite the majority of the women not attending school past primary years, the majority chose to birth in a health facility with a skilled health professional rather than at home with a TBA.

Instead of formal education, it seemed that women were receiving information about their health, especially antenatal care and reproductive health, from their neighbors and village health workers. The women in Kibibi seemed aware of the possibility of complications, which influenced their decision about where to deliver. Women were able to incorporate their learning into decision-making around birth place. All the women attended antenatal care at least once, which also contributed to seeking a skilled birth attendant, similar to the study conducted in northern Uganda in 2015 [12]. The attitude to delivery with a skilled birth attendant in Kibibi is indicative of the importance of community wide health conversations on individual behavior and choice. These conversations become the means through which health behavior is shared, conveyed and ingrained into the community [26].

### Transport and funds

Previous studies have highlighted lack of funds and transport as factors limiting access to health care facilities [11, 12, 16]. For the women of Maligita, the presence of a skilled attendant in the village helped to overcome those factors that leave women vulnerable. Issues of funds and lack of transport were less pressing, especially in low-risk births that didn’t require referral to hospital care. This highlights the importance of locally accessible services.

### Cultural competence

It is important that health professionals who provide ante-natal and birthing care have an understanding of cultural competence and beliefs around birth [16, 28]. In the case of Kibibi the local registered midwife CM had both an understanding of local culture, and the skills and experience in handling complications. The Process of Cultural Competence in the Delivery of Health Care Services by health professionals is a model proposed by Campinha-Bacote, [28]. This model incorporates the five constructs - cultural awareness, cultural knowledge, cultural skills, cultural desire, and cultural encounters. Within this model, it is possible for health professionals to develop cultural competence through a commitment to integrate the five constructs [29]. Campinha-Bacote contends that the main construct that leads to cultural competence is *Cultural Desire*: where different approaches to health care are sought and is the foundation for the other constructs. *Cultural Knowledge*: where there is knowledge of other cultural beliefs and health practices; *Cultural Awareness*: is a willingness to learn about other worldviews; *Cultural Skills*: is the ability to conduct holistic health assessments; and *Cultural Encounters* that are necessary to build real-world experiences.

For the women interviewed it seemed that visiting a TBA was a last resort, a back-up plan in the event of not being able to reach a health centre or hospital or lacking the funds to do so. This was especially prevalent at night, when the nearest health centre, is closed and it is difficult to get transportation. Despite previous studies highlighting the acceptability of TBAs due to their cultural awareness and willingness to facilitate valued cultural practices at birth [11, 12], this preference can be overcome if the locally available skilled birth attendant also demonstrate cultural competence.

### Lack of emergency care

In both Maligita and Kibibi, women had access to a skilled birth attendant, however, women who experienced complications during delivery still needed to be referred to a hospital where options were lacking and that equipment, and supplies such as blood and oxygen, are readily available. For the women of Maligita, CM would refer them and even accompany them to hospitals. At times CM used her own money to pay for a woman to take a motorcycle taxi to the nearest hospital, 30 km away. She even told the story of a woman who was put in a wheelbarrow and pushed to the road where she could get transport to the hospital. Whilst a valuable service, this fragility of access is not sustainable, and needs to be addressed.

For the women of Kibibi, the Health Centre IV could help with normal deliveries, however, some problems could only be handled at a hospital due to lack of staffing, severity of complications and inadequate equipment at the health centre. The Health Centre IV closest to Kibibi is equipped with an ambulance. However, the women are responsible for paying for their own fuel, which is beyond their economic means. Therefore, even though proximity can assist in overcoming many obstacles that cause delays in seeking care, problems will continue to arise if emergency obstetric care is not an easily available to women who need it. Additionally it appears that the women and men in this study were conscious of both safety and respect and TBAs were generally the least preferred option for these families when given a safer option. Much progress is needed to ensure that all women have timely access to all levels of maternal health care.

The study provided some rich data from a small group of participants in two villages however there are some limitations to the study. The sample was small and thus it is not possible to generalise the findings, however the recommendations proposed may be transferable to other similar settings in Uganda and Africa. The time and funding constraints meant that more villages could not be included in the study. Although the authors had all lived in Uganda, they did not speak the local language, however all precautions were taken through use of trained community interpreters who were bilingual. These villages had the advantage of a qualified midwife CM who spoke the local language and English.

Qualitative research is judged in terms of its dependability, credibility, confirmability and transferability [30] and trustworthiness is an important component as it demonstrates reliability and validity [31–33]. To enhance the credibility and overall trustworthiness of this study, we employed a range of techniques such as systematic checking, and ongoing interpretation of data [22, 34]. An audit trail ensured that information relating to the study context, methodology, data analysis and results were well documented so that the study could be replicated by other researchers [35, 36]. Our aim was to also provide sufficient interpretative to make judgments possible and to facilitate transferability of the study [30, 37, 38] by providing detailed descriptions.

### Recommendations drawn from participants

In 2016, the WHO developed Standards for Improving Quality of Maternal and Newborn Care in Health Facilities as part of the Sustainable Development Goals [39]. However, guidance on how these standards can be put into effect is scarce as evidenced by a content analysis of 123 WHO guidelines by Wang, Norris and Bero [40]. Hence, based on the findings from the focus groups and key informants some recommendations for policy makers can be offered.

The participants shared that a process of community consultation and problem solving be undertaken to explore the following - the need for availability of community funds that could overcome obstacles for pregnant women in labour thereby creating a safety net for vulnerable women unable to save for themselves or in the event of unforeseen complications. Training current health care workers in woman-centred care in order to change attitudes and enhance the provision of dignified patient care as a means of encouraging attendance to health facilities. A dialogue between health care workers, the community and patients could serve to bridge issues of contention. This could be initiated through professional development in patient communication and care [41]. Move from a one-size fits all approach since, as seen in this small study, each community has its own set of strengths and weaknesses. The empowerment of men and women through grassroots health education, has been shown to have an impact on maternal and child mortality and morbidity in developing countries [39, 40]. Empowering communities to identify and leverage its strengths, recognize its needs and work to find solutions is critical.

## Conclusions

Entrenched social and cultural factors create major barriers to the achievement of reproductive health and safe delivery for women especially in the developing world [42]. This study demonstrated that women value their own safety and the safety of their babies. Acceptability is a strong determining factor in where a woman will choose to give birth. While availability is clearly an important factor, women will choose to go further away if they perceive a better quality service. When a good quality service is close by, community members will use it, but when the services are not to be trusted, they will be bypassed. This study also notes that women and men are aware of the importance of accessing a qualified health professional at the time of birth. The challenges identified in this exploratory study highlight the need for more research into the community maternal health needs and the need for more culturally appropriate strategies for the provision of reproductive and safe childbirth. Many of the social factors affecting women’s health also affect the entire community and health programming needs to incorporate interventions that involve the community and include educational, economic and culturally appropriate components. It is our hope that this study will add to the growing body of research in understanding the complex issues of maternal health in resource poor settings.

## Data Availability

The datasets used and/or analysed during the current study are available from the authors on reasonable request.

## Abbreviations

CHW: Community Health Worker
CM: Certified Midwife
FGDs: Focus Group Discussions
TBA: Traditional Birth Attendant
UNPF: United Nations Population Fund
VHT: Village Health Team
WHO: World Health Organization
